# Cardioprotective effects of dapagliflozin against the acute cardiotoxic effects of 5-fluorouracil

**DOI:** 10.3389/fcvm.2025.1633420

**Published:** 2025-07-18

**Authors:** Mehmet Hakan Uzun, Sebahat Ulusan, Afragül Yönet, Kadir Şeker, Murat Sevimli, Kanat Gülle, Adnan Karaibrahimoğlu, Aliye Kuyumcu, Selçuk Kanat, Mehmet Alagöz, Mevlüt Serdar Kuyumcu

**Affiliations:** ^1^ Department of Cardiology, Republic of Türkiye Ministry of Health Kutahya City Hospital, Kutahya, Türkiye; ^2^ Department of Histology and Embryology, Suleyman Demirel University Health Sciences Institution, Isparta, Türkiye; ^3^ Department of Histology and Embryology, Suleyman Demirel University Faculty of Medicine, Isparta, Türkiye; ^4^ Department of Cardiology, Suleyman Demirel University Faculty of Medicine, Isparta, Türkiye; ^5^ Department of Histology and Embryology, Eskisehir Osmangazi University Faculty of Medicine, Eskisehir, Türkiye; ^6^ Department of Biostatistics and Medical Informatics, Suleyman Demirel University Faculty of Medicine, Isparta, Türkiye; ^7^ Department of Nutrition and Dietetics, Suleyman Demirel University Faculty of Health Sciences, Isparta, Türkiye; ^8^ Cardiology and Cardiac Electrophysiology, Private Clinic, Bursa, Türkiye; ^9^ Department of Surgery, Nassau University Medical Center, East Meadow, NY, United States

**Keywords:** 5-fluorouracil, cardiotoxicity, heart failure, SGLT2 inhibitors, cardiomyopathy

## Abstract

**Background:**

5-Fluorouracil (5-FU) has potential cardiotoxic effects, including direct cardiomyocyte damage, arrhythmia development, and coronary vasospasm. Numerous studies have demonstrated that dapagliflozin (DAPA) possesses cardioprotective properties. Theoretically, DAPA may have the potential to mitigate 5-FU-induced cardiotoxicity.

**Methods:**

32 male Wistar albino rats were randomly divided into four groups of eight animals each: Control, DAPA, 5-FU, and 5-FU + DAPA. The 5-FU groups received a single intraperitoneal dose of 150 mg/kg 5-FU at the beginning of the study, while the DAPA groups were administered 10 mg/kg DAPA daily via oral gavage. Echocardiography, electrocardiography, and weight measurements were performed at baseline, and at the end of the first and second weeks. The experiment was concluded at the end of the second week, and tissue samples were collected for histopathological analysis.

**Results:**

Compared to the 5-FU group, the 5-FU + DAPA group exhibited a 9.5% smaller reduction in ejection fraction, a 50% lower incidence of ST-segment elevation, and a 14.16% smaller increase in heart rate. Moreover, the prolongation of PR, QTc, and QRS durations was attenuated by 8.27%, 9.91%, and 34.5%, respectively (*p* < 0.05 for all). Histopathological analysis also revealed significantly reduced inflammation in the 5-FU + DAPA group (*p* < 0.05).

**Conclusions:**

Dapagliflozin has shown to have cardioprotective effects against acute cardiotoxicity in a 5-FU-induced cardiomyopathy rat model.

## Highlights

1. In this study, we have found that the 5-FU group experienced a 9.5% more decrease in ejection fraction, compared with the 5-FU + DAPA group. While ejection fraction values remained >50% in both groups, deterioration difference was found to be statistically significant in the 5-FU group.
2. Cardiac tissue histological evaluation showed that the 5-FU + DAPA group has significantly less inflammatory response, compared with the 5-FU group.
3. Deterioration in ECG parameters (PR, QRS and QTc durations) and development of ST segment elevation was found to be statistically significantly higher in the 5-FU group, compared with the 5-FU + DAPA group.

## Background

5-Fluorouracil (5-FU) has been widely used for many years in the treatment of various malignancies due to its potent antitumor effects. Although generally well tolerated, 5-FU can exert toxic effects on the cardiovascular system (1, 2). Its cardiotoxicity has been associated with the development of heart failure, cardiomyopathy, myopericarditis, arrhythmias, and sudden cardiac death (2). Several mechanisms have been proposed to explain this toxicity, including coronary endothelial dysfunction, coronary vasospasm, increased oxidative stress, direct cellular damage, and myocardial inflammation (3). Numerous experimental studies have aimed to prevent 5-FU-induced cardiotoxicity; however, no specific therapeutic regimen has been established to date (4–6).

Dapagliflozin (DAPA) is the first-line treatment recommended by current guidelines for the treatment of heart failure, regardless of ejection fraction. It has been shown to reduce the risk of cardiovascular death and hospitalization for heart failure in the DECLARE-TIMI 58, DAPA-HF, and DELIVER studies (7–9). The molecular mechanisms underlying these cardioprotective effects are primarily attributed to changes in intracellular Ca^2+^ handling and antioxidant effects; however, no definitive mechanism has been identified to this date (10, 11). Although several studies have reported significant cardioprotective effects of SGLT2 inhibitors at the histopathological level against various cardiotoxic agents, to the best of our knowledge, no study has comprehensively assessed their protective effects across histopathologic, electrocardiographic, and echocardiographic parameters (12–15). Addressing this gap, we aimed to evaluate the cardioprotective effects of DAPA against 5-FU induced cardiotoxicity in an experimental animal model, with a particular focus on structural, electrical, and functional cardiac changes.

## Methods

### Animal experiment method

The study included 32 male Wistar albino rats, housed under controlled conditions (22–24°C; 12 h light/dark cycle) with *ad libitum* access to food and water. After a 10-day acclimatization period, the rats were randomly assigned to four groups (*n* = 8 per group): Control, DAPA, 5-FU, and 5-FU + DAPA. Male rats were selected to minimize hormonal variability. The study was approved by the Süleyman Demirel University Animal Experiments Local Ethics Committee (Approval No: 09/105, dated 22.12.2022), in accordance with CPCSEA guidelines under the Prevention of Cruelty to Animals Act, 1960, and conducted in line with the Universal Declaration of Animal Rights.

**Figure 1:**
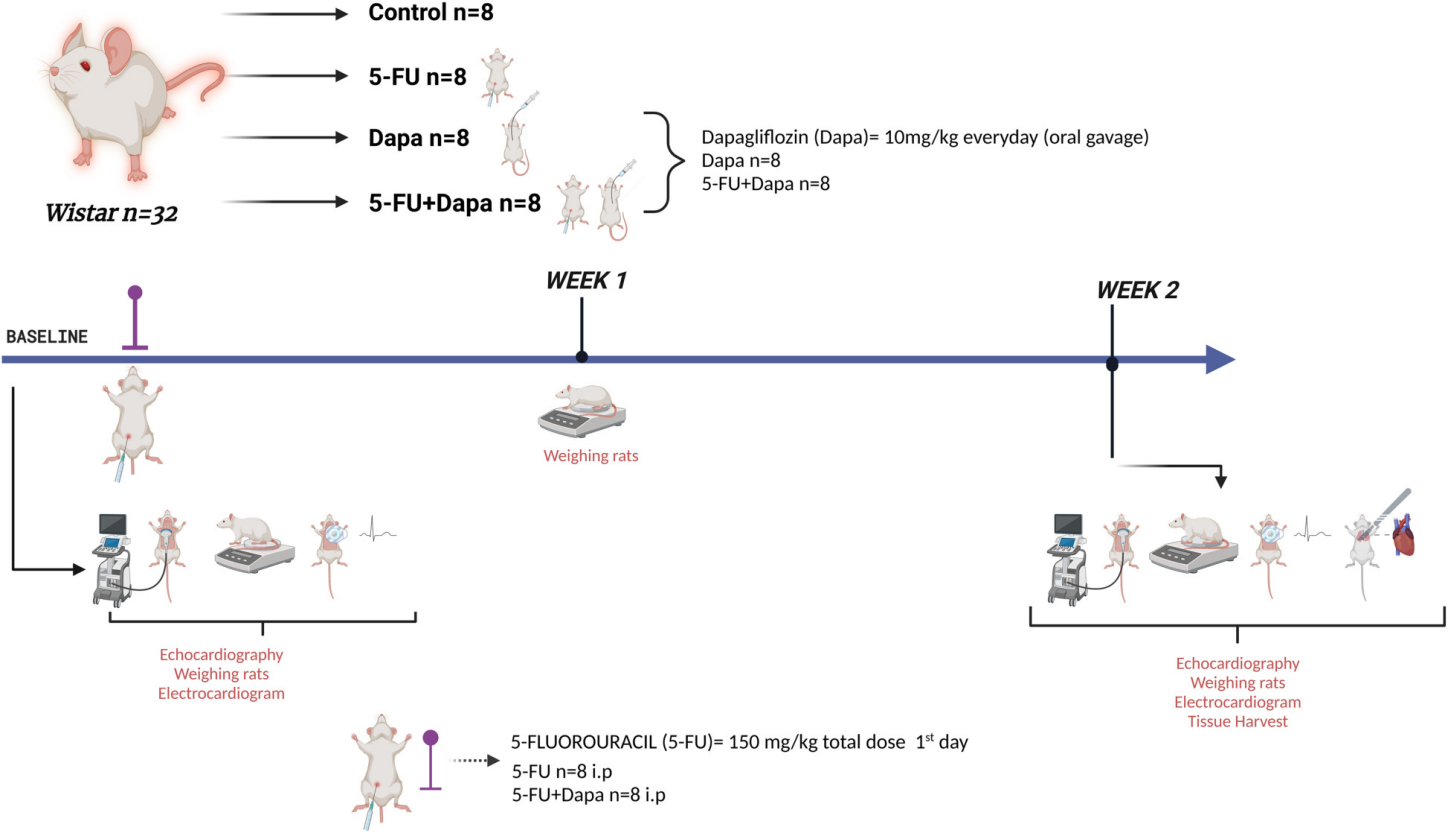
Experiment design. 5-FU 5-fluorouracil; DAPA, dapagliflozin.

Rats in the 5-FU and 5-FU + DAPA groups received a single intraperitoneal dose of 5-FU (150 mg/kg) at the start of the experiment. Rats in the DAPA-treated groups received 10 mg/kg of DAPA via oral gavage once daily for 14 consecutive days. The DAPA dose was selected based on current evidence and aligned with previously published literature (12–15). All animals underwent echocardiographic and electrocardiographic assessments at baseline and at the end of the second week. No mortality was observed during the study. The experimental protocol lasted 24 days in total, including a 10-day acclimatization period, and was concluded 14 days after 5-FU administration. At the end of the second week, anesthesia was induced intraperitoneally with ketamine (90 mg/kg) and xylazine (10 mg/kg). Anesthetic depth was monitored every two minutes by assessing jaw and skeletal muscle tone. Once adequate anesthesia depth was confirmed, the animals were sacrificed via exsanguination, a method chosen to preserve the structural integrity of cardiac tissues. Cardiotoxicity was further evaluated through histopathological analysis. The study design is visually summarized in Figure 1.

The sample size was estimated using the resource equation method for ANOVA, where E = total number of animals−total number of groups. With 32 animals distributed across 4 groups (*n* = 8 per group), E = 28, which exceeds the recommended range of 10–20 and thus indicates an adequate sample size. Increasing the number of animals further would not substantially improve statistical power.

No artificial intelligence assisted technologies were used in any part of this research.

### Electrocardiogram

The electrocardiograph machine (Fukuda Denshi Co. Ltd, Tokyo, Japan) was employed to conduct electrocardiograms (ECGs). Electrodes were positioned on the right wrist, sternum, right ankle, and left ankle of the anesthetized rats. ECGs were carried out both at baseline and at the conclusion of 2 weeks.

### Echocardiographic imaging

**Figure 2:**
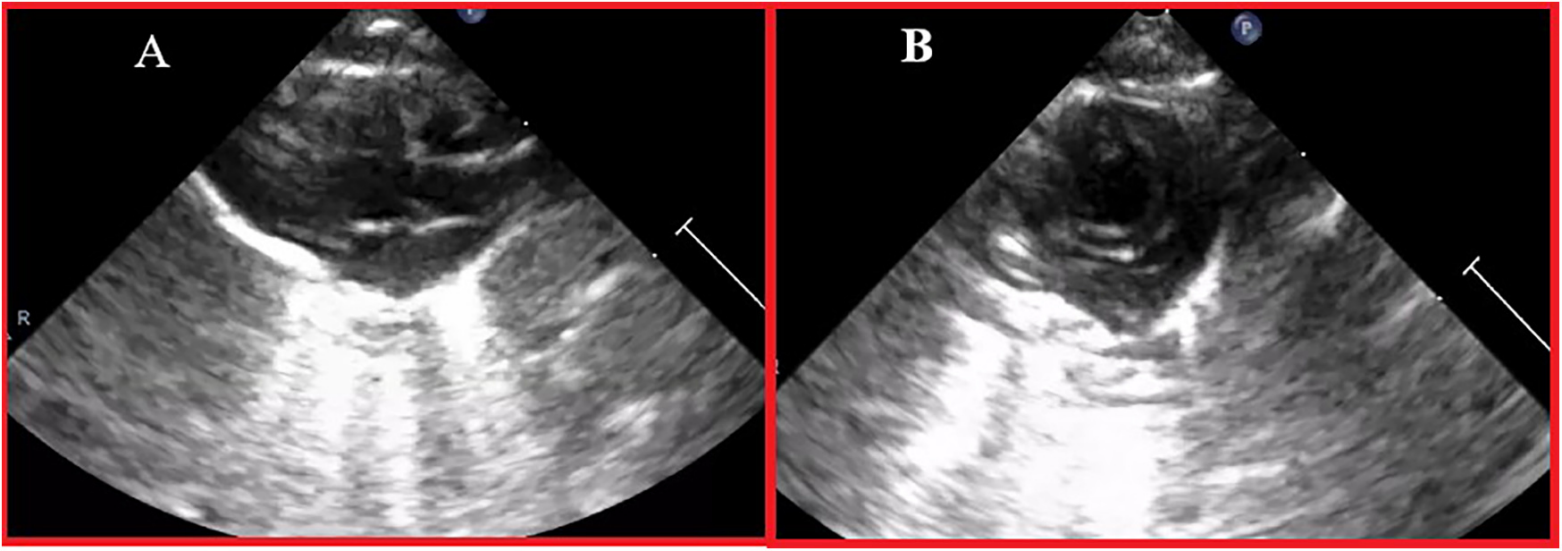
Echocardiographic image example. **(A)** Parasternal long axis view; **(B)** Parasternal short axis view.

Echocardiographic imaging was performed using the Philips Lumify system with a standard pediatric S4-2 transducer (Koninklijke Philips N.V., Amsterdam, Netherlands) on all animals at baseline (*n* = 32). All images were acquired using a standard echocardiography preset and were evaluated by two independent echocardiography operators. The zoom function was consistently set to 8× magnification across all evaluations. Each animal was imaged prior to receiving the initial dose of 5-FU and/or DAPA. Two weeks after treatment initiation, cardiac function was reassessed in all animals using two-dimensional echocardiography (Figure 2).

### Histopathological examination

Heart tissues were fixed in 10% neutral-buffered formalin, processed, and embedded in paraffin. Sections (4–5 μm) were stained with hematoxylin and eosin (H&E) following standard protocols, including deparaffinization, graded alcohol rehydration, hematoxylin staining, eosin counterstaining, dehydration, and xylene clearing. Slides were mounted with entellan and examined under a light microscope. Histopathological changes were scored as 0 (none), 1 (mild), 2 (moderate), or 3 (severe).

### Statistical analysis

Statistical analyses were performed using GraphPad Prism (v9). Data normality was assessed with the Kolmogorov–Smirnov test. One-way ANOVA with Tukey's *post hoc* test was used for group comparisons, and paired *t*-tests were applied for pre- and post-treatment analyses. Rat weights were evaluated using two-way repeated measures ANOVA. Variance homogeneity was assessed via Levene's test. Interobserver agreement for ECG and echocardiographic measurements was determined using the intraclass correlation coefficient (ICC). Results were expressed as mean ± SD, with statistical significance set at *p* < 0.05.

## Results

Four groups of 8 rats each were included in the study. One group served as the control group, while two groups received either DAPA or 5-FU treatment alone. The fourth group received a combination of 5-FU and DAPA. Efforts were made to ensure that all rats had comparable baseline body weights.

**Figure 3:**
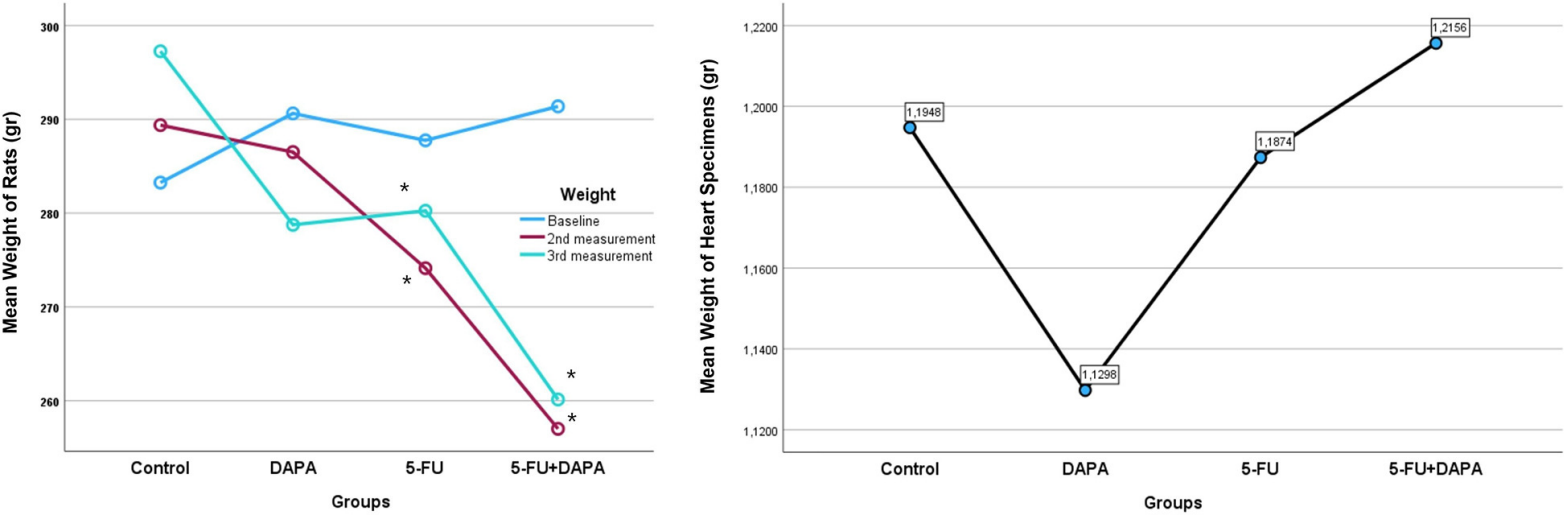
Body weight and heart weight changes. 5-FU, 5-fluorouracil; DAPA, dapagliflozin. *Significant at 0.05 level according to Two-way ANOVA.

No significant weight loss was observed in the DAPA and control groups; however, body weight was significantly reduced in the 5-FU and 5-FU + DAPA groups (*p* < 0.001). The treatments had no significant effect on heart weight, and there were no statistically significant differences in heart weights among the groups at the end of the experiment (*p* = 0.643) (Figure 3).

**Figure 4:**
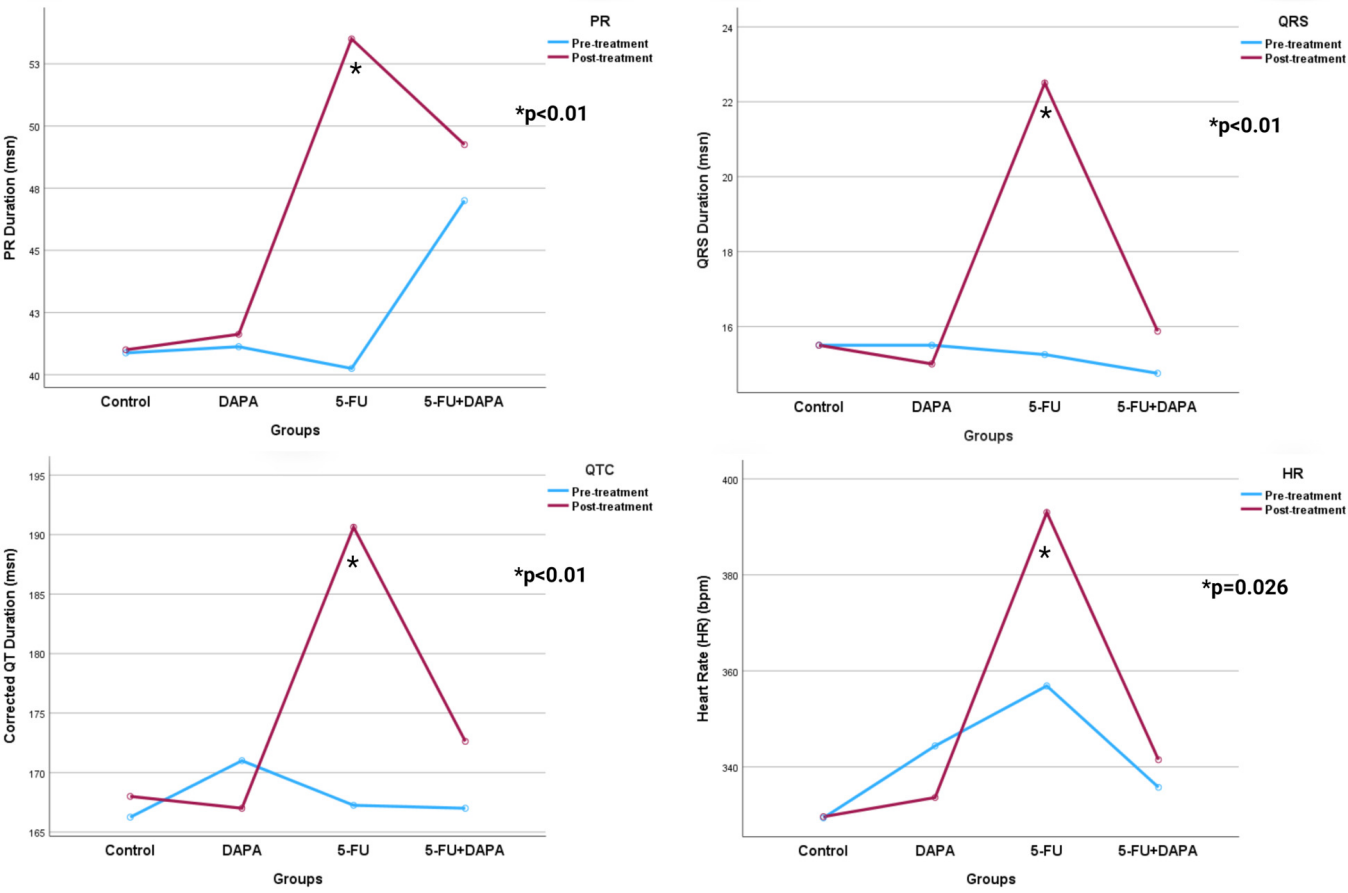
Electrocardiogram parameters. 5-FU, 5-fluorouracil; DAPA, dapagliflozin. *Significant at 0.05 level according to Two-way ANOVA.

While no ST-segment elevation was observed in any group at baseline, post-treatment ST elevation occurred in 75% of rats in the 5-FU group and in 25% of rats in the 5-FU + DAPA group (*p* = 0.031). Although the 5-FU group exhibited higher baseline heart rates compared to the other groups, a statistically significant increase in heart rate was observed in this group at the end of the treatment period (*p* < 0.001). QTc intervals did not differ significantly between groups at baseline. However, in the 5-FU group, the QTc interval increased significantly from 167.25 ± 15.82 ms at baseline to 190.62 ± 21.36 ms post-treatment (*p* < 0.001). QTc prolongation was significantly reduced in the 5-FU + DAPA group compared to the 5-FU group (*p* = 0.001). A significant increase in PR interval was also observed in the 5-FU group after treatment (53.50 ± 6.23 ms) compared to baseline (40.25 ± 3.19 ms) (*p* < 0.001). Although the baseline PR interval in the 5-FU + DAPA group was higher than in the other groups, the increase in PR duration following treatment was significantly lower compared to that observed in the 5-FU group (*p* = 0.007). Additionally, the mean QRS duration in the 5-FU group increased significantly from 15.25 ± 1.98 ms at baseline to 22.50 ± 3.81 ms post-treatment (*p* < 0.001), while no significant QRS changes were observed in the other groups (Figure 4).

**Figure 5:**
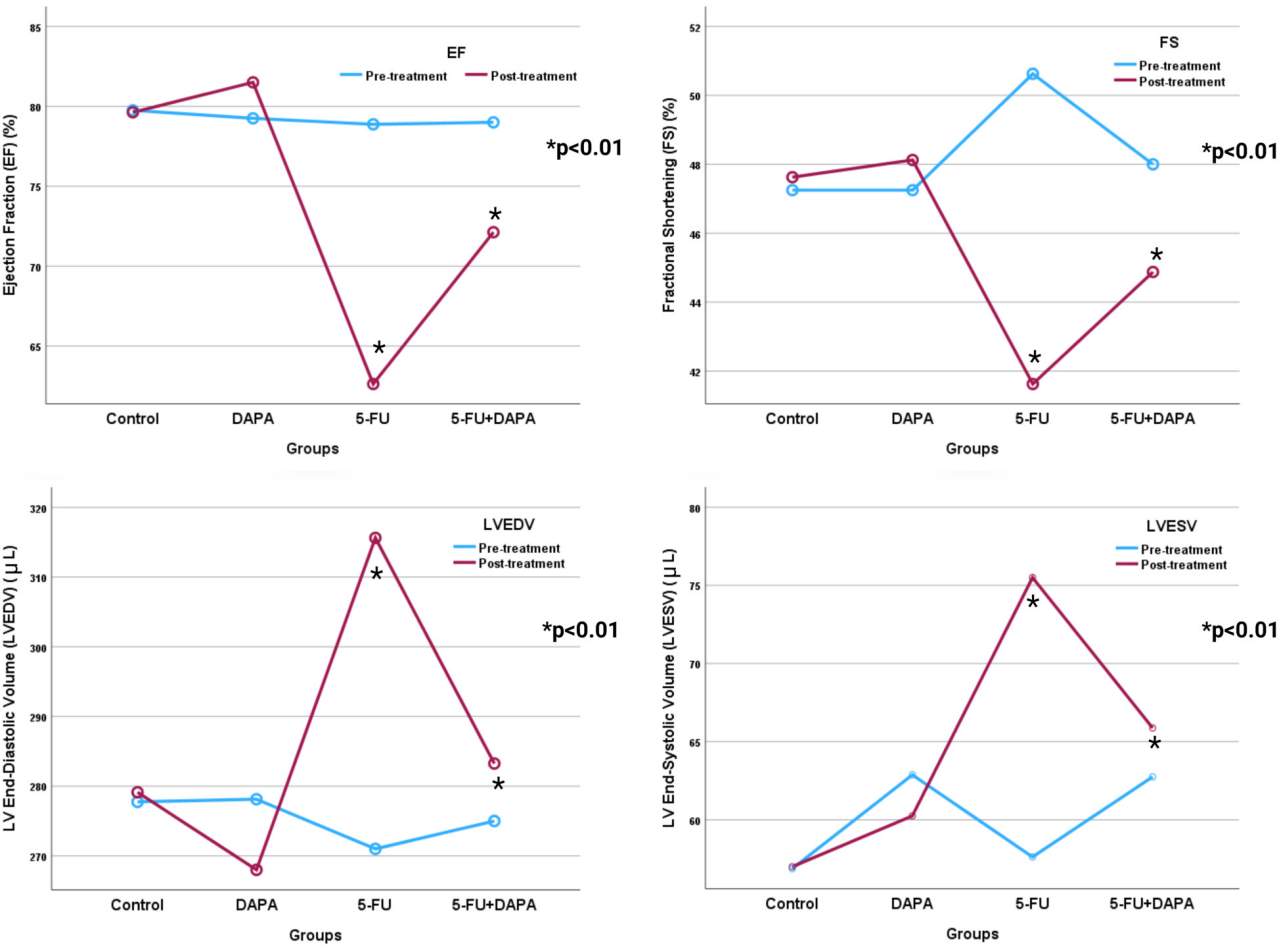
Echocardiographic parameters. 5-FU, 5-fluorouracil; DAPA, dapagliflozin. *Significant at 0.05 level according to Two-way ANOVA.

Left ventricular systolic ejection fraction (EF) and fractional shortening (FS) values declined significantly more in the 5-FU group compared to the 5-FU + DAPA group (62.62 ± 6.32% vs. 72.12 ± 6.08%, and 41.62 ± 4.47% vs. 44.87 ± 3.44%, respectively; *p* < 0.001). Additionally, although baseline measurements showed no statistically significant differences, the 5-FU group exhibited significantly greater increases in left ventricular end-systolic diameter (LVESD) and end-diastolic diameter (LVEDD) compared to the 5-FU + DAPA group (315.62 ± 35.28 µl vs. 283.25 ± 15.43 µl, and 75.50 ± 11.16 µl vs. 65.87 ± 8.79 µl, respectively; *p* < 0.001) (Figure 5). No statistically significant changes were observed in the control or DAPA groups across any echocardiographic parameters. A summary of all echocardiographic findings is presented in Table 1.

**Table 1:** Cardiac and electrocardiographic measurements of animal study groups.

Parameters	Control	DAPA	5-FU	5-FU + DAPA	*P*_groups_	*P*_measures_
	Mean ± SD	Mean ± SD	Mean ± SD	Mean ± SD		
First weight measurement, g	283.25 ± 28.71	290.62 ± 26.46	287.75 ± 36.84	291.37 ± 26.02	*0*.*521*	*<0.001* * *1–2* *1–3*
Second weight measurement, g	289.37 ± 46.94	286.50 ± 24.16	274.12 ± 26.43	257.00 ± 23.82		
Third weight measurement, g	292.25 ± 40.57	278.75 ± 22.44	280.25 ± 23.08	260.12 ± 17.06		
Ejection fraction, baseline, %	79.75 ± 1.03	79.25 ± 1.83	78.87 ± 2.35	79.00 ± 3.25	*<0.001* * *a–c* *a–d* *b–d*	*<0*.001*
Ejection fraction, week 2, %	79.62 ± 1.59	81.50 ± 2.92	62.62 ± 6.32	72.12 ± 6.08		
Fractional shortening baseline, %	47.25 ± 3,80	47,25 ± 5.23	50.62 ± 4.62	48.00 ± 4,30	*0*.*848*	*<0*.001*
Fractional shortening week 2, %	47.62 ± 3.85	48.12 ± 5.51	41.62 ± 4,47	44.87 ± 3,44		
End-diastolic volume baseline, μl	277.75 ± 12,41	278.12 ± 10.99	271.00 ± 13.26	275.00 ± 15.00	*0*.*083*	*0*.*001*
End-diastolic volume week 2, μl	279.12 ± 11.93	268.00 ± 14.24	315.62 ± 35,28	283.25 ± 15.43		
End-systolic volume baseline, μl	56.87 ± 10,13	62.87 ± 4.83	57.62 ± 7,26	62.75 ± 8.53	*0*.*132*	*<0,001* *
End-systolic volume week 2, μl	57.00 ± 10,40	60.25 ± 6.04	75.50 ± 11,16	65.87 ± 8.79		
Heart rate baseline, bpm	329.37 ± 23.76	344.37 ± 27.51	358.87 ± 12.95	337.75 ± 20.80	*0.002* * *a–c* *b–c* *d–c*	*0*.026*
Heart rate week 2, bpm	329.62 ± 25.25	336.62 ± 23.37	393.00 ± 34.94	341.50 ± 22.97		
QTc baseline, ms	166.25 ± 18.52	171.00 ± 16.54	167.25 ± 15.82	167.00 ± 13.00	*0*.*849*	*<0*.001*
QTc week 2, ms	168.00 ± 18.05	167.00 ± 15.47	190.62 ± 21.36	172.62 ± 14.12		
PR baseline, ms	40.87 ± 5.16	41.12 ± 5,22	40.25 ± 3.19	47.00 ± 5.37	*0.007* * *a–d* *b–d*	*<0*.001*
PR week 2, ms	41.00 ± 5.01	41.62 ± 4.59	53.50 ± 6.23	49.25 ± 5.49		
QRS baseline, ms	15.50 ± 1.77	15.50 ± 1.60	15.25 ± 1.98	14.75 ± 1.28	*0.001* * *a–c* *b–c* *d–c*	*<0*.001*
QRS week 2, ms	15.50 ± 1.77	15.00 ± 1.30	22.50 ± 3.81	15.87 ± 1.26		
Heart weight, g	1.19 ± 0.17	1.12 ± 0.11	1.18 ± 0.10	1.21 ± 0.15	*0*.*643*	
ST elevation, *N* (%)	0 (0%)	0 (0%)	6 (75.0%)	2 (25.0%)	*0*.031**	

* Significant at 0.05 level according to Two-way ANOVA.

** Significant at 0.05 level according to Chi-square test.

5-FU, 5-fluorouracil; DAPA, dapagliflozin.

**Figure 6:**
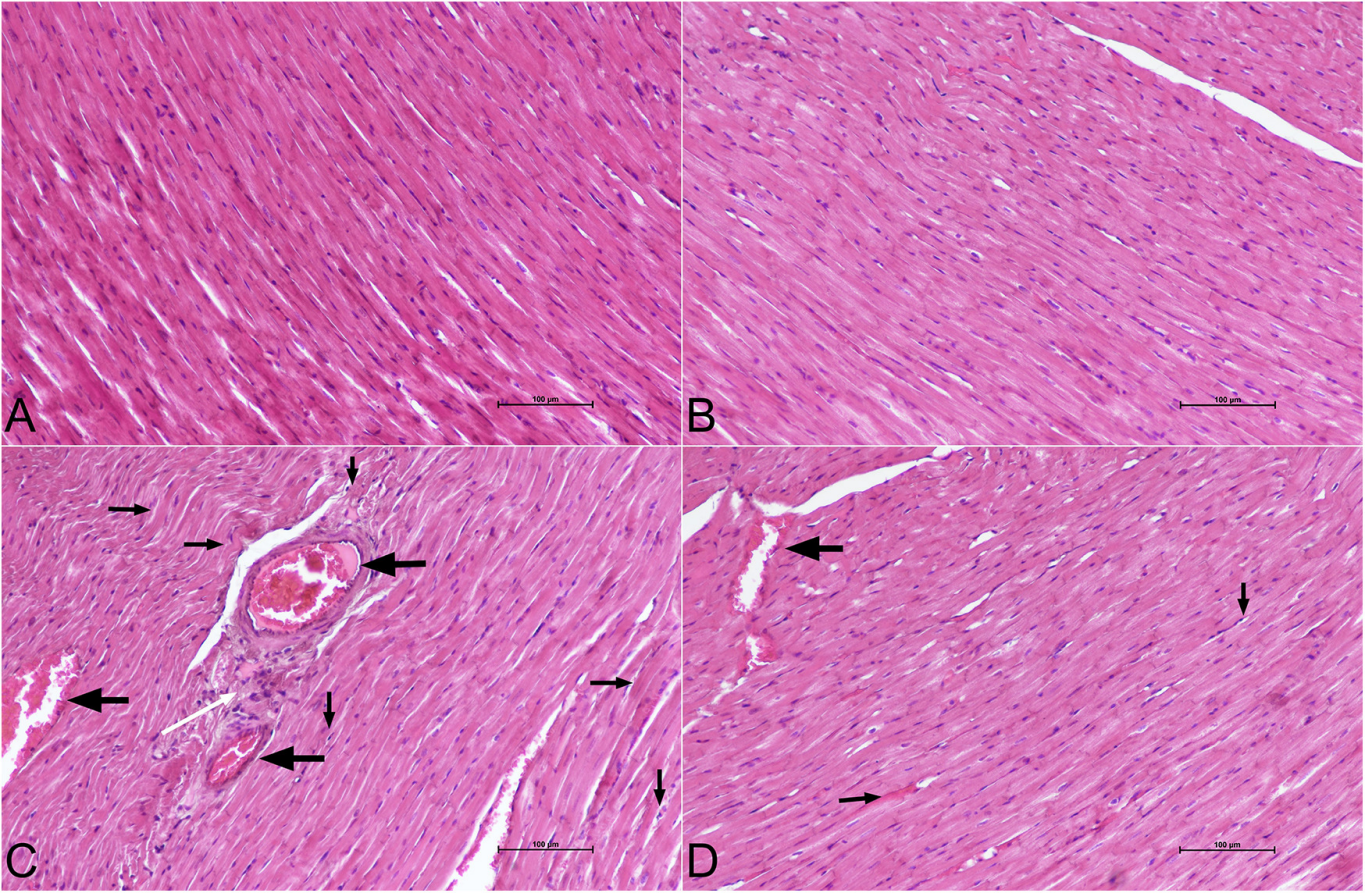
Histopathological figures. Histopathological findings of cardiac tissue. **(A)** Control group: normal cardiac tissue histology. **(B)** DAPA group: normal cardiac tissue histology **(C)** 5-FU group: hyperemia (left arrows), necrosis (down arrows), inflammatory cell infiltrations (white arrow) and hyaline formation (right arrows) **(D)** 5-FU + DAPA group: hyperemia (left arrows), necrosis (down arrows) and hyaline formation (right arrows). H&E staining, ×200, scale bar = 100 µm. 5-FU, 5-fluorouracil; DAPA, dapagliflozin.

The H&E staining results showed that the cardiac tissue histology was normal in control (Figure 6A) and DAPA groups (Figure 6B). On the other hand, the 5-FU group showed high levels of cardiotoxicity and severe histopathological findings, including hyperemia, necrosis, inflammatory cell infiltration, and hyaline formation compared with the control group (Figure 6C) (*p* < 0.001 for all findings). Significantly less inflammatory response was observed in the 5-FU + DAPA group in histopathological findings compared to the 5-FU group (Figure 6D) (*p* < 0.001 for all findings). All the findings and histopathological scores are summarized in Table 2.

**Table 2:** Histopathological examination results of animal study groups.

Parameter	Experimental groups			
	Control	DAPA	5-FU	5-FU + DAPA
Hyperemia	0 ± 0^a^	0.142 ± 0.378	2.857 ± 0.378^a,b^	0.571 ± 0.534^b^
Necrosis	0.285 ± 0.488^a^	0.285 ± 0.488	2.571 ± 0.534^a,b^	0.857 ± 0.378^b^
Inflammatory cell infiltration	0 ± 0^a,b^	0.142 ± 0.378	2.714 ± 0.488^a,c^	0.714 ± 0.488^b^^,^^c^
Hyaline formation	0 ± 0^a^	0 ± 0	2.429 ± 0.534^a,b^	0.428 ± 0.534^b^

Values are expressed as mean ± SD.

5-FU, 5-fluorouracil; DAPA, dapagliflozin.

^a,b,c^ Same superscript letters denote the statistically significant differences at 0.05 level across pairwise groups according to Tukey's HSD *post hoc* test.

## Discussion

The cardiotoxicity of 5-FU has been attributed to several mechanisms, including coronary vasospasm and direct cellular damage, with clinical manifestations ranging from mild elevations in troponin levels to overt ventricular dysfunction (16). The cardiac conduction system is also vulnerable to 5-FU–induced toxicity, potentially leading to arrhythmias of varying severity (17).

Signs of coronary vasospasm include ST-segment changes on electrocardiography and elevated serum troponin levels in the absence of visible coronary artery occlusion on invasive coronary angiography. Vasospasm can be triggered by various mechanisms, including endothelial dysfunction and the subsequent cascade of reactions that disrupt the balance between vasoconstrictive–vasodilative and prothrombotic–antithrombotic pathways. Dysfunction of coronary arterial smooth muscle cells is another proposed mechanism, which may result from the direct cellular toxicity of 5-FU. The resulting vasospasm leads to myocardial hypoxia and consequent cardiac injury. Although coronary vasospasm is considered one of the primary mechanisms underlying 5-FU–induced cardiotoxicity, conflicting evidence exists in the literature; not all patients with 5-FU–related cardiomyopathy exhibit documented or inducible vasospastic responses (16).

5-FU induced direct cellular damage plays an important role in the development of cardiotoxicity. It has been shown that 5-FU induces mitochondrial decoupling, which results in reduced aerobic metabolism and subsequent hypoxia in cardiomyocytes (18). In result, reactive oxygen species (ROS) are produced, which cause increased oxidative stress and reduced levels of antioxidants, due to depletion. Several studies have shown consistent findings with this theory (19, 20). Even though several mechanisms are proposed for the explanation of cardiotoxicity, the most possible conclusion is that all of these mechanisms are inter-linked and each plays an important role. Thus, a holistic approach covering all mechanisms in targeting 5-FU induced cardiotoxicity is needed.

Numerous clinical trials have shown that SGLT2 inhibitors have potent cardioprotective effects, which in turn reduces cardiovascular adverse events, including heart failure hospitalizations and cardiovascular mortality (7–11, 21, 22). Anti- oxidant and anti-inflammatory effects of SGLT2 inhibitors have also been shown in numerous trials (23–25). Normalization in tissue ROS/antioxidant molecule balance may have a critical role in the prevention of 5-FU induced cardiotoxicity. Due to the fact that cardiomyocytes do not express SGLT2, cardioprotective effects of SGLT2 inhibitors are thought to be mediated by other signaling pathways. In an *in vitro* experiment by Chen et al. (12) has shown that DAPA alleviates hypoxia/reoxygenation induced cardiomyocyte damage by reducing intracellular Fe^2+^ concentration by reducing PTGS2 levels and promoting SLC7A11/GPX4 axis expression via the MAPK pathway, thus reducing ROS generation and cardiomyocyte damage. Several studies on this subject have also yielded similar results, which suggests MAPK pathway plays a crucial role in cardioprotective effects of SGLT2 inhibitors (26, 27).

To date, no experimental studies in humans have investigated the cardioprotective effects of SGLT2 inhibitors against specific cardiotoxic agents, such as radiotherapy or chemotherapy. Numerous experimental studies in animal models have been undertaken to explore this area. In a study conducted by Mahmoud Refaie et al. (28), the cardioprotective effects of DAPA against cyclophosphamide-induced cardiotoxicity were evaluated. The findings demonstrated that DAPA conferred significant cardioprotective benefits. Notably, the combination of DAPA and cyclophosphamide resulted in increased immunoexpression of endothelial nitric oxide synthase (eNOS) and vascular endothelial growth factor (VEGF) compared with the cyclophosphamide group, suggesting enhanced endothelial function and angiogenic activity. In a study by Lahnwong et al. (29), the acute administration of dapagliflozin (DAPA) was shown to exert cardioprotective effects against ischemia/reperfusion injury by reducing the incidence of arrhythmias, limiting infarct size, and attenuating cardiac apoptosis. Similar to our study, an experimental animal model by Refaie et al. (15) investigated the cardioprotective effects of Empagliflozin against 5-FU induced cardiotoxicity and reported comparable findings. Rats administered 5-FU alone exhibited significant upregulation in the expression levels of nuclear factor kappa B (NF-κB), interleukin-1β (IL-1β), interleukin-6 (IL-6), myeloid differentiation primary response 88 (MYD88), tumor necrosis factor-alpha (TNF-α), SGLT2, p53, and caspase-3, compared with the Empagliflozin + 5-FU group. Although structural changes were not specifically evaluated in that study, the reported histopathological findings are consistent with those observed in our research.

In our study, we have found statistically significant differences in ST-segment elevation development; changes in LVEDD, LVESD, heart rate, durations of PR, QRS, and QTc between groups which receive 5-FU and 5-FU + DAPA. While the reductions in EF and FS values were statistically more significant in the 5-FU group compared to the 5-FU + DAPA group, the mean EF values in both groups remained above 50%. Additionally, LVEDD and LVESD values increased in both groups but were statistically significantly lower in the 5-FU + DAPA group in comparison with 5-FU group. Correllation between echocardiographic and histopathological findings in our study are consistent with the notion that reducing inflammation and limiting the progression of cardiac fibrosis help preserve myocardial microarchitecture, thereby contributing to the maintenance of normal cardiac functions. In humans, clinical trials have demonstrated that SGLT2 inhibitors exert significant effects in preserving LVEF, irrespective of baseline LVEF levels (7–9), and our findings in the experimental model are consistent with these clinical observations. Nonetheless, further research is needed to investigate the effects of SGLT2 inhibitors on cardiac function in patients undergoing 5-FU therapy.

Alterations in heart rate, as well as the durations of PR, QRS, and QTc intervals, also suggest potential anti-arrhythmogenic effects of DAPA, likely mediated by its ability to reduce myocardial tissue disruption and preserve conduction system integrity (13, 14). These findings from our experimental animal model align with human clinical evidence; notably, *post-hoc* analyses of the DAPA-HF trial demonstrated that DAPA was associated with a 21% reduction in the risk of serious ventricular arrhythmias, resuscitated cardiac arrest, or sudden cardiac death compared with placebo (8).

Histopathological examination revealed that the 5-FU + DAPA group exhibited significantly less hyperemia, hyaline formation, inflammatory cell infiltration, and necrosis, findings that are closely associated with a reduced degree of acute cardiotoxicity. The cardioprotective effects observed are thought to be mediated by the pleiotropic actions of SGLT2 inhibitors, including their anti-inflammatory, antioxidant, and metabolic regulatory effects on cardiomyocytes (15, 26–30).

Although the severity of the acute inflammatory response is linked to the development of chronic changes, this process is multifactorial, and efforts to develop new screening methods to identify patients at higher risk of 5-FU-induced cardiotoxicity are ongoing. Given the cardiac anti-fibrotic effects of SGLT-2 inhibitors demonstrated in HF patients, as well as in experimental animal modes, we believe further research is needed in this area to explore potential therapies for preventing 5-FU-induced cardiotoxicity.

It should not be forgotten that we were not able to investigate inflammatory and cardiac biomarkers in our study, as well as changes in the diastolic functions and strain analysis patterns, which are the main limitations of our study. With no exact molecular mechanism of cardioprotective effects of SGLT2 inhibitors has been shown to this date, further detailed research in this area is required to shed light on the molecular basis of cardioprotective effects of SGLT inhibitors.

## Conclusions

Our study has shown potential cardioprotective effects of DAPA against 5-FU cardiotoxicity. However, concomitant possible side effects such as decreased appetite and loss of weight in cancer patients may prove to have restrictive use of DAPA, due to the weight losing effects of SGLT2 inhibitors. Careful evaluation of nutritional status and benefit-loss ratio is needed to avoid deterioration in clinical status.

## Limitations

In our study, we were not able to assess ventricular strain patterns and diastolic functions of rats to compare cardiotoxic effects of 5-FU in ventricular strain patterns and diastolic parameters, and potential beneficial effects of SGLT2 inhibitors against it. Second, 5-FU was injected intraperitoneally in our study, causing direct liver damage. This is not the routine clinical use of 5-FU, and its intravenous use may yield different results than our findings. Third, we were not able to examine biochemical parameters of inflammation and/or molecular mechanisms. Finally, it is unclear whether a cardioprotective effect can be achieved in rats with cancer, since our study used healthy rats only.

## Data Availability

The raw data supporting the conclusions of this article will be made available by the authors, without undue reservation.
